# Electrospun fibers of zein and pea protein to create high-quality fibrous structures in meat analogs

**DOI:** 10.3389/fbioe.2024.1483966

**Published:** 2024-10-28

**Authors:** Letícia G. da Trindade, Letícia Zanchet, Fabiana Perrechil Bonsanto, Anna Rafaela Cavalcante Braga

**Affiliations:** ^1^ Department of Chemical Engineering, Campus Diadema, Universidade Federal de São Paulo (UNIFESP), Diadema, Brazil; ^2^ LRC - Institute of Chemistry, Universidade Federal do Rio Grande do Sul (UFRGS), Porto Alegre, Brazil; ^3^ Department of of Biosciences, Campus Baixada Santista, Universidade Federal de São Paulo (UNIFESP), Santos, Brazil

**Keywords:** plant protein, electrospinning, fibers, plant-based, innovative food

## Abstract

**Introduction:**

The importance of developing plant-based meat similar to animal meat lies in the fact that sensory similarity is a crucial factor in encouraging consumers to adopt this alternative.

**Methodology:**

The present study reports the morphology, hydrophilicity, and thermal analysis of different fibers obtained by the electrospinning method. In the first step of this work, zein and zein/poly(ethylene oxide) (PEO) in 80% aqueous ethanol solution with varying concentrations of these polymers were investigated.

**Results and Discussion:**

It was observed that the diameters of the electrospun fibers are related to the concentration and viscosity of the solutions. Moreover, the addition of small percentages of PEO makes the fibers more hydrophilic and leads to an increase in the polymeric solution viscosity. Because of its low toxicity, PEO is used in various edible products. In the second step of this work, an ideal zein/PEO combination was found to allow the pea protein (PP) to be electrospun. Adding PP to the zein/PEO blend (20:1) leads to a more hydrophilic fiber and improves thermal stability. The results suggest that the zein/PEO and zein/PEO/PP blends can offer an innovative solution to enhance the texture and appearance of plant-based meats. These simulated electrospun fibers can mimic the fibers in animal meat and are a potential alternative to provide a sensory experience as close to animal meat as possible.

## 1 Introduction

Biomaterials have recently emerged as effective solutions for improving food industry by utilizing their unique characteristics to enhance new food products, ingredients, packaging, preservation, and quality control in the food sector. A key focus area is the creation of food systems based on biomaterials. Several innovative biomaterials are viewed as viable substitutes for ingredients in foodstuff as well as in packaging due to its biodegradable and renewable properties. It offers numerous benefits, such as favorable mechanical characteristics, lower production costs, and lesser environmental impact compared to traditional materials. Modern biomaterials for food application include chitosan, carboxymethyl cellulose, starch, pea protein, and wheat gluten. These materials, composed of carbohydrates (polysaccharides) or proteins (polypeptides), have demonstrated their efficacy in combating food uses (Halder et al., 2024).

Recently, it has become increasingly evident that the meat industry needs to be more sustainable, as raising livestock requires many resources and contributes to releasing greenhouse gases into the atmosphere. In this context, the search for sustainability increases the interest in vegetable proteins. Since it requires fewer resources for its production, there is a considerable decrease in the greenhouse effect and the reduction of other environmental impacts, such as conserving land and water (Scarborough et al., 2014). This beneficial environmental impact and the fact that the necessary source of protein in the human diet is not given up when consuming vegetable proteins increases the search for meat analogs. These products are mainly composed of thickened, texturized, or extruded proteins and try to mimic the sensory qualities of meat (van Esbroeck et al., 2024).

Despite the great interest in meat analogs, there still needs to be a significant gap in the market, as accurately mimicking meat’s characteristic orientation (fibrous structure) has proven difficult. Of the currently available meat analogs (Pietsch et al., 2019), extrusion or high-temperature shearing produces the most meat-like structures (Dekkers et al., 2016). However, these methods have obstacles and limitations to the final product’s size based on the equipment’s size (which limits the size of the piece produced), high-cost equipment, and the need for a high-energy input (Liang et al., 2019).

These barriers to producing meat analogs can be overcome by developing methods to create a fibrous material from plant proteins using inexpensive, less energy-intensive methods. Among the different structuring techniques to create fibrous plant protein materials that can be used to develop meat analogs, we can highlight electrospinning. This technique adopts a bottom-up strategy, creating anisotropic structural elements later assembled into more oversized products (Forgie et al., 2023).

In electrospinning, a polymer solution is pushed through a hollow needle with an electric potential relative to a ground electrode. Accumulation of charge on the droplet’s surface that emerges from the spinneret causes surface instabilities that ultimately grow into fibers attracted to the ground electrode (Schiffman and Schauer, 2008).

Electrospinning fibers can be produced from a variety of solutions, including plant proteins such as zein and polymers such as poly (ethylene oxide) (PEO). Fibers obtained by this technique have unique characteristics, such as high surface area, porosity, and good mechanical properties, making them suitable for various biomaterial applications. Fibers made from zein or blends of zein and PEO can produce biomaterials with enhanced properties compared to pure polymers. Specific applications of zein or zein/PEO fibers include tissue engineering (Pérez-Guzmán and Castro-Muñoz, 2020), controlled drug delivery (Surendranath et al., 2023), biomedical implants (Medeiros et al., 2021), and biomedical sensors (Zdraveva et al., 2023).

However, the electrospinning of vegetable proteins has its challenges since, to apply the electrospinning technique efficiently, the protein must be highly soluble and behave like a random coil instead of globulins (Dekkers et al., 2018). These requirements are often not met by plant proteins, such as pea protein, since they are in their native globular state and, when denatured, form insoluble aggregates. An exception presented in the literature is zein, the primary storage protein from corn, which accounts for 35%–60% of the total proteins of corn and is found exclusively in the endosperm (Assad et al., 2020). Zein is highly hydrophobic, meaning it has no affinity for water and tends to clump together into a water-insoluble structure. However, when treated with organic solvents, it can dissolve and become a spinnable complex. Therefore, while most globular proteins are not spinnable due to their compact, hydrophobic structure, zein can be electrospun due to its ability to become charged when dissolved in appropriate solvents. This plant protein can be electrospun in ethanol (80 wt. %) (Ramos et al., 2022).

Adding small quantities of poly (ethylene oxide) (PEO) is an alternative to provide elasticity to the formation of fibers combined with zein and other proteins. PEO is a hydrophilic polymer safely used in food fields due to its non-toxicity, biocompatibility, and biodegradability (Lin et al., 2017; Khoshnoudi-Nia et al., 2020). Moreover, among the polymers reported in the literature, PEO is biodegradable, has a high molecular weight, and is certified as Generally Recognized as Safe (GRAS) (FDA UNII 16P9295IIL) and has been approved by the Food and Drug Administration (FDA) (Ramos et al., 2020; 2021). Hence, it can be safely used in processed food and beverages.

This research aimed to produce fibers using the electrospinning method from zein and polymeric blends of zein/PEO to use fibrous structures in meat analogs. In addition, aiming to increase the protein content and variety in these fibers, an ideal zein/PEO combination was chosen, and pea protein was added to the formulations. Obtaining pea protein fiber has limitations, such as low protein concentration, low structural stability, inadequate rheological properties, electrochemical compatibility, and technological limitations (Wei et al., 2020). Therefore, combining with other proteins may be an efficient strategy to overcome these limitations and obtain electrospinning fibers containing pea protein.

Merging zein/PEO and pea protein to generate electrospun fibers for plant-based meats could be an exciting and promising area of research in the vegetarian and vegan food technology field. Zein has film-forming and gelling properties, while pea protein is rich in essential amino acids and has gelling properties (Wei et al., 2020). The combination of these two proteins together with low percentages of PEO can lead to the formation of fibers that resemble the texture and appearance of muscle fibers present in conventional meat. The advantage of combining these three materials is that zein/PEO can help improve the adhesion and stability of the fibers, preventing separation during processing and cooking. Furthermore, the presence of pea protein enriches the nutritional profile of zein/PEO fibers, providing an additional source of essential amino acids.

## 2 Materials and methods

### 2.1 Materials and solution preparation

Zein, ethanol (99.8%), and poly (ethylene oxide) (PEO) (900,000 g moL^−1^) were supplied by Sigma-Aldrich. Pea protein concentrate (Pea Standard 80 ST0845, protein content 82.8% dry basis) was provided by Gramkow (Joinville, Brazil).

Zein solutions (12%, 20%, and 33% w/v) were prepared by dissolving different amounts of pure zein in 80% ethanol and maintained under constant stirring for 1 hour at room temperature. The polymeric blend solution with varying proportions of zein/PEO was prepared similarly to the pure zein solution except for slowly adding 0.3 or 1% (w/v) PEO. All the studied formulations of zein and zein/PEO and their nomenclature designations are shown in Table 1.

**TABLE 1:** Sample composition, sample designation, mean of the results, the standard deviation, the Tukey test for the rheological parameters, and macroscopic and microscopic observations during and after the spinning process for 80% ethanol aqueous solutions of different zein and zein/PEO concentrations.

Sample composition (%)	Sample designation	k (Pa.s^n^)	n (−)	R^2^	Viscosity at 10 s^−1^ (mPa.s)	Visual observation at needle	Microscopic observation on collector
Zein 12	Z12	0.0148^c^ ± 0.000	0.980^a^ ± 0.017	99.99	20.0^b^ ± 10.7	jet	short fibers
Zein 20	Z20	0.0439^c^ ± 0.001	0.960^a^ ± 0.007	99.98	39.2^b^ ± 0.78	jet	fibers
Zein 33	Z33	0.454^bc^ ± 0.118	0.845^bcd^ ± 0.042	99.93	236.3^b^ ± 17.80	jet	fibers
Zein 12% PEO 0.3	Z12P03	0.0311^c^ ± 0.009	0.941^a^ ± 0.008	99.99	26.3^b^ ± 7.71	jet	fibers
Zein 12% PEO 1	Z12P1	0.2676^c^ ± 0.066	0.811^b^ ± 0.017	99.96	135.7^b^ ± 25.6	jet	fibers
Zein 20% PEO 0.3	Z20P03	0.0959^c^ ± 0.003	0.927^abc^ ± 0.001	99.99	73.5^b^ ± 2.08	jet	fibers
Zein 20% PEO 1	Z20P1	0.5442^b^ ± 0.033	0.814^bc^ ± 0.003	99.93	286.9^b^ ± 23.7	jet	fibers
Zein 33% PEO 0.3	Z33P03	1.0656^ab^ ± 0.623	0.861^ab^ ± 0.120	99.31	1,448.6^a^ ± 593.7	jet	fibers
Zein 33% PEO 1	Z33P1	1.7483^a^ ± 0.422	0.829^bc^ ± 0.025	99.95	1,200.5^a^ ± 414.9	jet	fibers

Equal letters indicate no significant difference between the means (Tukey test, p> 0.05) in the same column.

Pea protein/zein/PEO solutions were prepared by adding pure pea protein (PP; 1, 2, 3, or 5% w/v) in distilled water and stirring for 10 min at room temperature. Then, this solution was heat-treated at 80°C for 30 min under stirring to denature the protein. At the same time, the pure zein (20% w/v) was dissolved in 80% ethanol under magnetic stirring for 1 h. With the two solutions ready, they were mixed at the predetermined concentrations. After this time, 1% PEO was added to facilitate the formation of electrospun nanofibers.

### 2.2 Rheological analysis of solutions

Rheological characterizations of solutions were carried out on Anton Paar MCR 92 Rheometer (Anton Paar, Austria) at 25°C ± 0.2°C. Samples were loaded under a 25 mm or 50 mm (depending on the viscosity of the solution) parallel plate with a 1 mm gap. An up-down-up step program was applied with the shear rate varying from 0.1 s^-1^ to 300 s^-1^. Rheological parameters were obtained by fitting the Power Law model (Equation 1) to the flow curves at the steady state.

$$\sigma =k\cdot {\dot{\gamma }}^{n}$$ (1)

where σ is the shear stress (Pa), k is the consistency index (Pa.s^n^), n is the flow behavior index (−), and $\dot{\gamma }$ is the shear rate (s^−1^).

### 2.3 Electrospinning process

The homogeneous polymer solutions were loaded into a 5 mL syringe attached to a tip of 1.03 mm internal diameter, and the electrospinning was made using a laboratory-scale electrospinning machine (FLUIDNATEK LE-10, BIOINICIA, Spain). The process and solution parameters for the electrospinning process were chosen based on a previous study on nanofiber production (Ramos et al., 2022). The voltage of 22 kV, tip-to-collector distance of 15 cm, and flow rate of 3,000 μL/h parameters were used to fabricate zein, zein/PEO, and pea protein/zein/PEO fibers. The nanofibers were deposited on a rotatory collector, at 150 rpm, with an aluminum foil of 13 × 20 cm size at controlled room temperature (20°C–25°C) and relative humidity (50%–60%).

### 2.4 Fiber’s characterization

The nanofibers’ morphology was studied by JEOL JSM-6610LV scanning electron microscope (SEM) (JEOL, Japan) operating at 10 kV. The ImageJ software was used to determine the size distribution of fiber diameters; 100 randomly selected points in the SEM images were selected. The ATR-FTIR spectra (Nicolet 6700 model equipped with a germanium crystal in ATR mode) of nanofibers were used to investigate the possible structural interactions in the samples. The spectra were collected over the 4,000–650 cm^−1^ wavenumber range with 128 scans. The thermal stability of the fibers was evaluated by TGA (Discovery -TA equipment) with a flow of 60 mL min^−1^ of ultra-pure nitrogen as a purge gas, and the decomposition was analyzed in the range from 25°C to 700°C, with a heating rate of 10°C min^−1^. Fiber hydrophilicity was evaluated by measuring the contact angle of a water drop placed over the sample using a DataPhysics OCA 11 goniometer at 25°C.

### 2.5 Statistical analysis

The rheological parameters were analyzed using the One-Way ANOVA, followed by Tukey as a *post hoc* test, and the Jamovi software. The significant differences (p> 0.05) between means were then identified.

## 3 Results and discussion

### 3.1 Results of the rheological analysis of solutions

Several fiber formulations were successfully manufactured by combining the proposed matrices. As reported in the literature, the present work confirms that electrospinning is affected by polymer solution parameters and processing conditions (Mendes et al., 2017; Hosseini and Mahdi, 2020). As expected, changing these parameters can influence the electrospinning process and the electrospun fiber morphology. Solution viscosity is a factor affected by polymer concentration and, together with the solution’s electrical properties, will determine the extent of the solution’s elongation. These two factors, in turn, affect the diameter of the electrospun fiber (Park et al., 2008).

**FIGURE 1:**
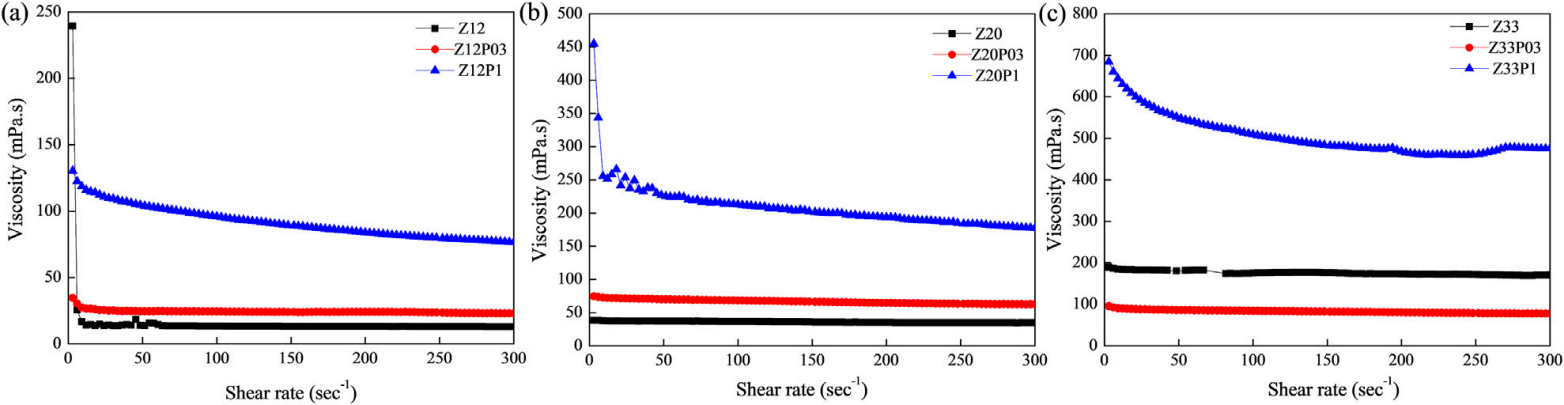
Viscosity is a function of the shear rate of the viscosity for 80% ethanol aqueous solutions of different zein and zein/PEO concentrations: **(A)** samples Z12, Z12P03 and Z12P1; **(B)** samples Z20, Z20P03 and Z20P1; **(C)** samples Z33, Z33P03 and Z33P1.

Figure 1 shows the flow curves of polymer solution for zein and zein/PEO with different concentrations in 80% ethanol aqueous solutions, and the rheological parameters are summarized in Table 1. It is clear from Figures 1A–C and Table 1 that increasing the concentration of both zein and PEO results in an increase, considering the solution’s viscosity is observed. This behavior can be attributed to the rise in the intertwining of polymer chains, thus increasing their interactions. However, with the increase in shear rate, there is a reduction in the viscosity of the solutions, indicating a shear-thinning behavior.

The values of the flow behavior index (n) for samples with 12% zein (0.970) and 20% zein (0.960), Table 1, were close to 1, suggesting that these samples demonstrate nearly Newtonian-like flow behavior. For the remaining samples, the value of n is less than 1, which confirms that these samples exhibit shear-thinning behavior. However, the Tukey test results indicate from the n values that in addition to the samples Zein 12% and Zein 20%, the samples Zein 12% PEO 0.3%, Zein 20% PEO 0.3%, and Zein 33% PEO 0.3% also demonstrate nearly Newtonian-like flow behavior in addition to shear-thinning behavior. It can also be observed from the results that the viscosity of all samples is similar except for the samples containing Zein 33% PEO 0.3% and Zein 33%PEO 1%, which are similar.

Still, from the rheological point of view, the consistency coefficient (k), a parameter linked to the apparent viscosity of materials across the entire range of shear rates, rises as the concentration of zein increases and with the addition of PEO. The coefficient obtained (R^2^) varied from 99.31 to 99.99, which confirms that the Power law model used provided a good fit for the flow curves. The uppermost viscosities and the notable shear thinning characteristics were observed for zein solutions containing 1% PEO. The shear thinning effect in this mixed solution may be due to the relaxation of PEO chains and the re-organization of zein along PEO chains. The behavior of larger polymer chains determines the viscosity profiles, as they significantly impact viscosity more than smaller proteins (Zhang et al., 2018).

The definition of the polymer solution composition and the process parameter to produce the microfibers was done based on the previous work of Ramos et al. (2022), who also studied electrospinning of the zein/PEO polymer blend. In this research, the authors evaluated the morphology and diameter of the fibers formed using a polymeric blend solution with 12% (w/v) of zein and two different PEO concentrations, 0.3% and 1% (w/v). The results showed that the solution of 12% zein/1% PEO produced electrospun fibers with a diameter of 201.3 ± 58.6 nm with the formation of homogeneous nanofibers in size and orientation.

In comparison, a composition of 12% zein/0.3% PEO, despite forming fibers with larger diameters (712.4 ± 415.1 nm), these fibers were not homogeneous and did not have a defined orientation. Each study can aim at fibers of distinct sizes; some applications require a microscale, and others require a nanoscale. In the case of the present work, the aim is to develop homogeneous fibers with a defined orientation to be applied in plant-based meat analogs. Still, it is crucial to obtain fibers that mimic animal muscle fibers possessing a diameter between 0.025 and 0.05 mm (Giménez-Ribes et al., 2024). To do so, we started our studies with 12% zein as a minimal polymer concentration. In addition, we changed the processing conditions to increase the diameter of the fibers obtained in the referred research by using a voltage of 22 kV and a feeding rate of 3,000 μL/h.

### 3.2 Fiber’s production and characterization

**FIGURE 2:**
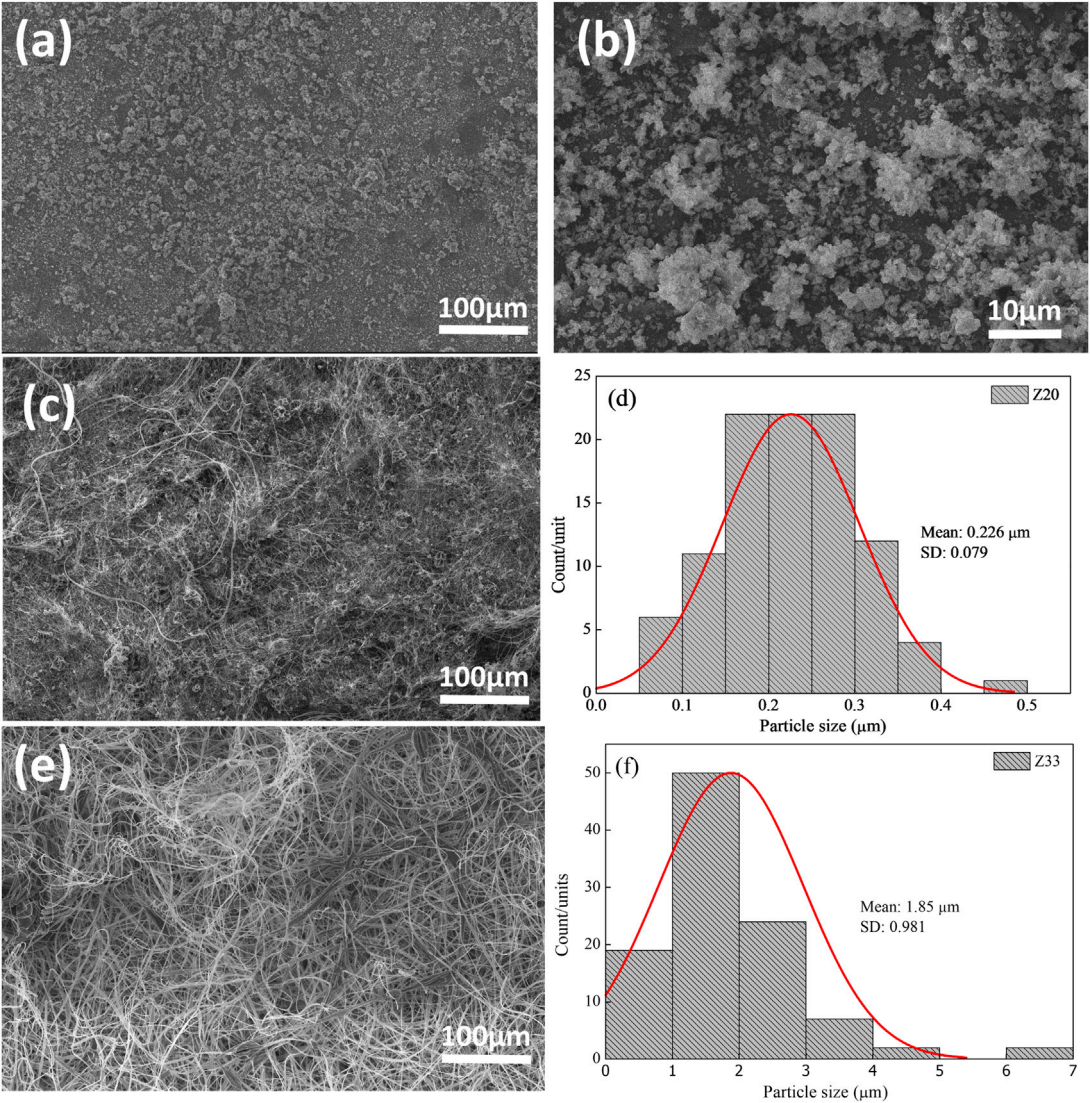
SEM images of Z12 **(A, B)**, Z20 **(C)**, Z33 **(E)**, and fibers diameter of Z20 and Z33 **(D)** and **(F)**, respectively.

**FIGURE 3:**
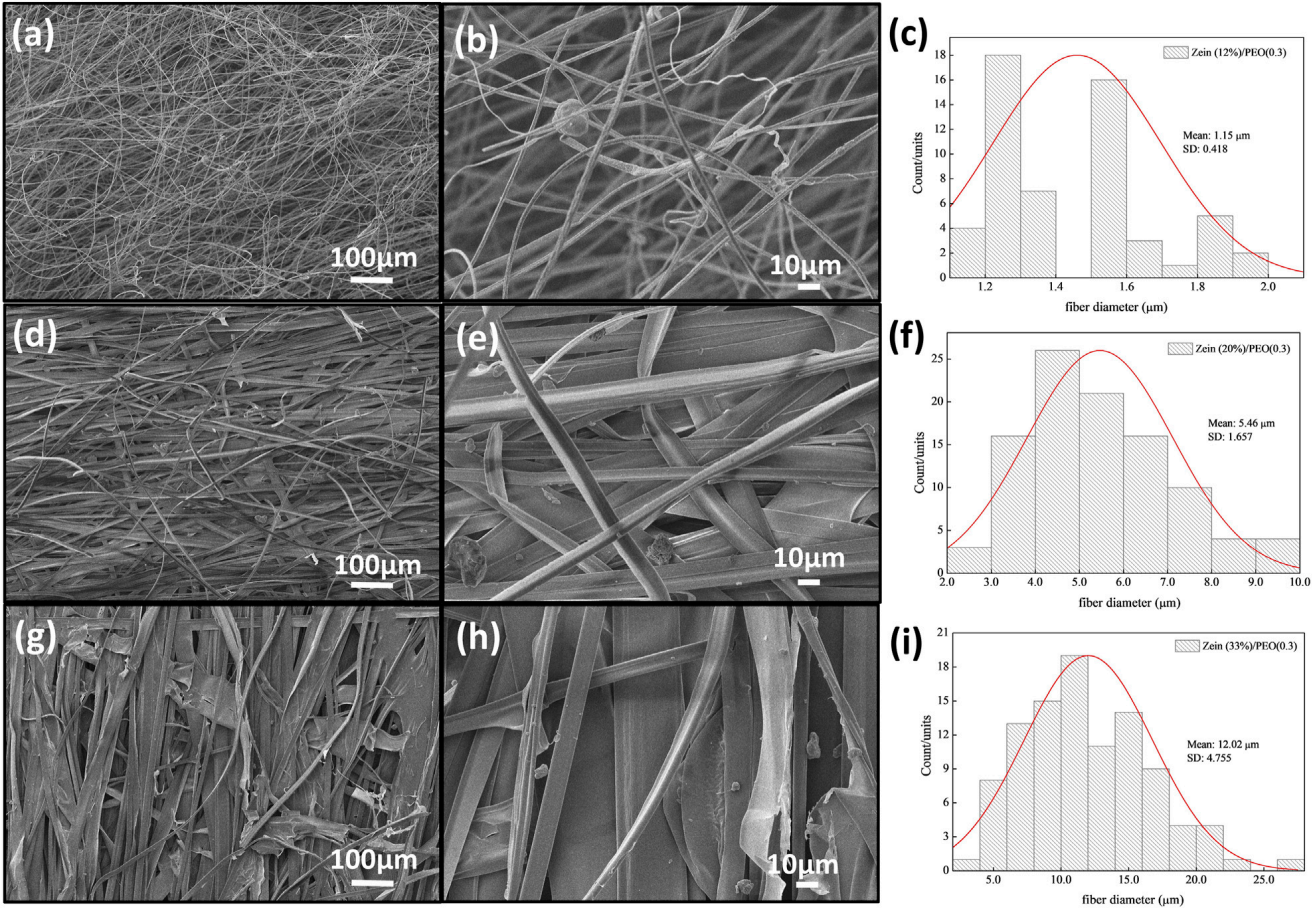
SEM images of Z12P03 **(A, B)**, Z20P03 **(D, E)**, Z33P03 **(G, H)** and their fibers diameter **(C)**, **(F)**, and **(I)**.

**FIGURE 4:**
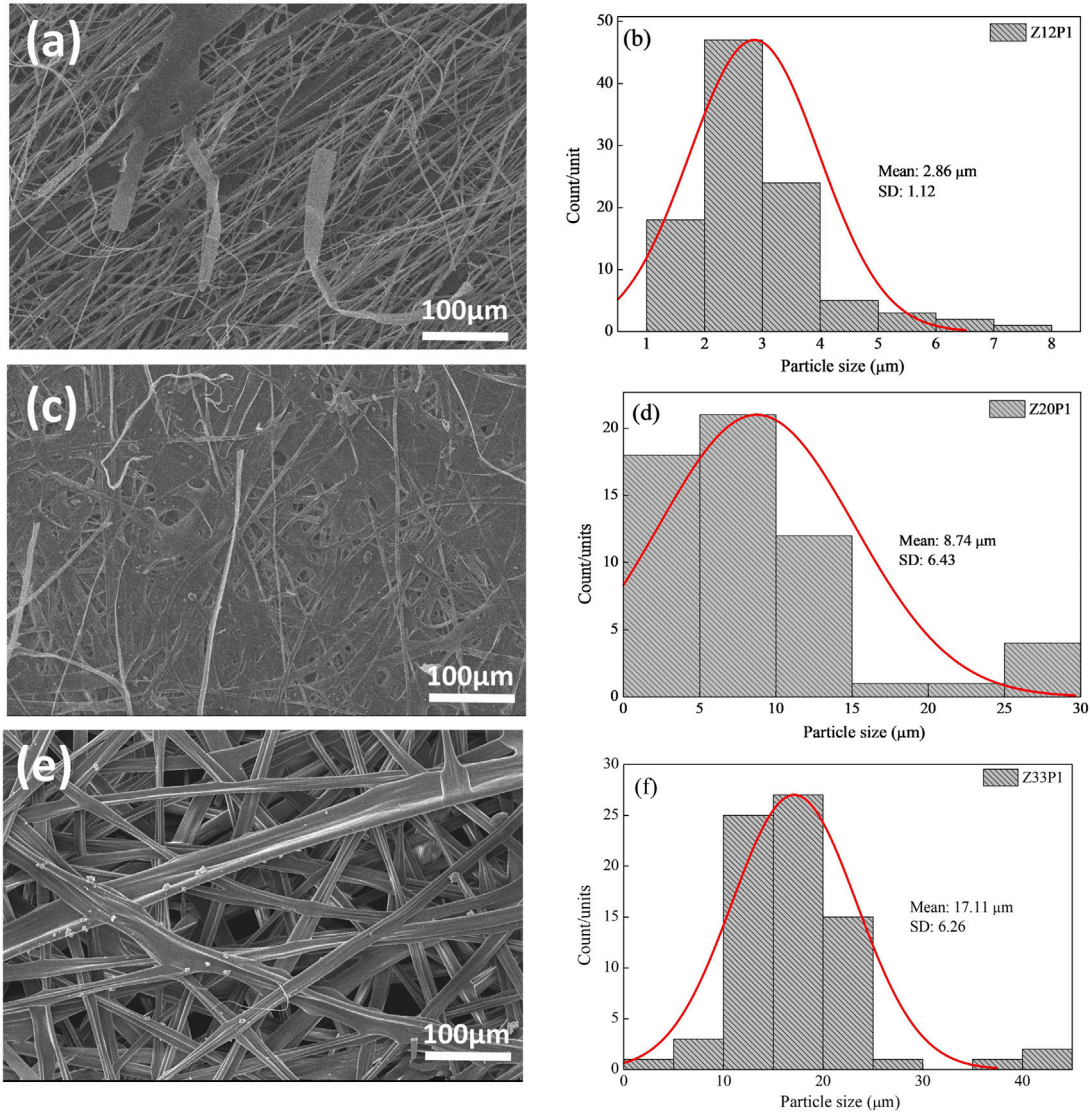
SEM images of Z12P1 **(A)**, Z20P1 **(C)**, Z33P1 **(F)** and their fibers diameter **(B)**, **(D)**, and **(F)**.

Figures 2–4 show the SEM images and the fiber diameters obtained by varying the zein concentration (12, 20, and 33% (w/v)) and with or without PEO addition in the samples in 80% (w/w) ethanol. Figures 2A, B presents the SEM images of fibers with 12% zein, which showed that, under these process conditions, the attempt at electrospinning fibers resulted in only droplets, probably due to the low solution viscosity. By increasing the zein concentration to 20% (w/v), Figure 2C, wrinkled microbeads and microfibers were produced. The zein microfibers obtained at this concentration are at sub-micro scale (width <1 μm) (Figure 2D). Figure 2E shows that an increase in the zein solution viscosity (33% (w/v)) led to the production of fibers with larger diameters than those in the other tested concentration (1.85 µm, Figure 2F) and without beads. On the other hand, adding PEO (0.3 wt.%), Figure 3, to the zein solution resulted in non-homogeneous fibers with varied diameters in the range of 1.15–12.02 µm (Figures 3C, F, I). This difference in the fiber’s appearance with PEO incorporation can be related to the PEO being a flexible and uncharged synthetic polymer. The PEO can decrease the repulsive force between polyanionic molecules by forming hydrogen bonds with zein and facilitating chain entanglement (Kyzioł et al., 2017).

By increasing the PEO concentration to 1% (w/v) in the zein solution, Figure 4, the fibers formed with 12% and 20% zein are irregular but with an average diameter higher than these zein solutions with 0.3% PEO, Figures 4B, D, F. This behavior can be attributed to the higher viscosity of the polymer solutions, which favors the formation of smooth fibers. It occurs because, in the electrospinning process, the surface tension of solutions drives the process toward the formation of beads. When the viscosity increases, bigger beads tend to be formed until the shape of the beads changes from spherical to spindle-like, producing the fibers (Fong et al., 1999). When the polymeric solution was increased to 33% zein and 1% PEO, the fibers reached their largest diameter with good homogeneity. The data confirms that adding PEO is necessary to generate bead-free fibers.

The results show that, for these formulations, the fibers’ morphology depended on the solution viscosity and the PEO addition. An increase in solution viscosity with increasing zein concentration contributes to the increase in the diameter of the fibers. The SEM images show that the rise in zein concentration, from 12% to 33%, favors the formation and growth in the fiber diameter. Adding PEO helped to form the fiber and contributed to the viscosity increase, favoring an increase in fiber diameter, one of our primary goals.

**FIGURE 5:**
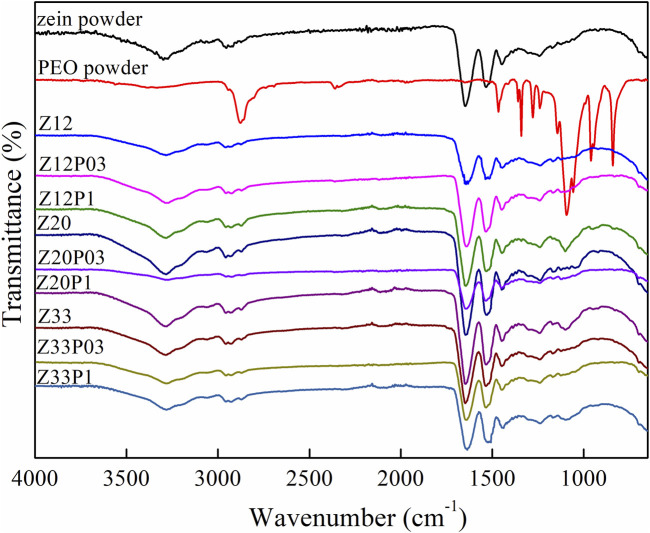
ATR-FTIR spectra of zein and PEO powders and electrospun fibers with different amounts of zein and PEO.

The ATR-FTIR spectra of the pure zein, pure PEO, and the electrospun fibers of Z12, Z20, and Z33 with the addition of 0.3% or 1% (w/v) PEO are presented in Figure 5. The associated effect of hydroxyl groups (O-H) that affects hydrogen bonding in the 3,290 cm^−1^ region of the films has been detected. Aliphatic compounds (C-H) contribute to the chain elongation in 2,940 cm^−1^. The characteristic bands that correspond to amide I, II, and III are presented at 1,642 (C=O stretching), 1,530 (N-H bending and C-N stretching), and 1,449 cm^−1^, respectively (Ali et al., 2014). For the pure PEO spectrum, it is possible to observe a broad band between 3,640 and 3,000 cm^−1^ that refers to O-H stretching vibrations. The band at 1,638 cm^−1^ can be attributed to the stretching vibration of the -CH_2_ group linked to the hydroxyl groups (Liu et al., 2019) and, at 1,091 cm^−1^, refers to the C-O-C ether linkage (Hegazy and Mahmoud, 2014). The ATR-FTIR spectra of the electrospun fibers of 12, 20, and 33 wt.% of zein (Z12, Z20, and Z33 samples) show spectra identical to that of pure zein, as expected.

However, when 1% PEO is added, the zein/PEO blends (Z12P1, Z20P1, and Z33P1 samples) show a 1,091 cm^−1^ band corresponding to the PEO ether C-O-C bond. An increase in the intensity of the bands in this region was observed, indicating the presence of PEO in the zein matrix. On the other hand, the FTIR spectra did not reveal the formation of new bands in the analysis of zein/PEO fibers, indicating no significant interactions between the materials. The compounds are just physically mixed, with no chemical reactions. Nevertheless, it is expected that the presence of these compounds directly interferes with the structural and functional properties of the fibers.

**FIGURE 6:**
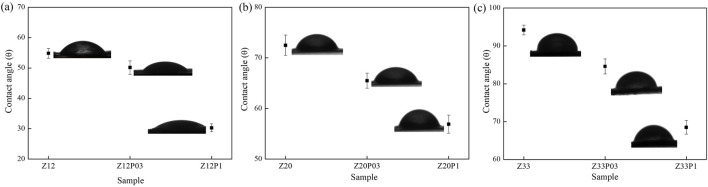
Water contact angle measurements of the electrospun zein fibers from samples: **(A)** Z12P03 (Zein 12% PEO 0.3%), **(B)** Z20 P03 (Zein 20% PEO 0.3%), and **(C)** Z33P03 (Zein 33% PEO 0.3%).

The water contact angles of the electrospun zein fibers without and with PEO addition are shown in Figure 6. Zein exhibits a hydrophobic character, increasing zein concentration in the fibers (Figures 6A–C). However, adding PEO in the two concentrations makes the fibers more hydrophilic (Figures 6A–C). The hydrophilicity of the fibers increases with increasing PEO concentration since this polymer is hydrophilic (Surendranath et al., 2022). To compose meat analogs, one of the most important characteristics of the fibers is hydrophilicity, as it is essential to guarantee the juiciness and softness of the final products. Furthermore, the hydrophilic fibers can hypothetically retain flavors and aromas, contributing to the consumer’s sensory experience.

**FIGURE 7:**
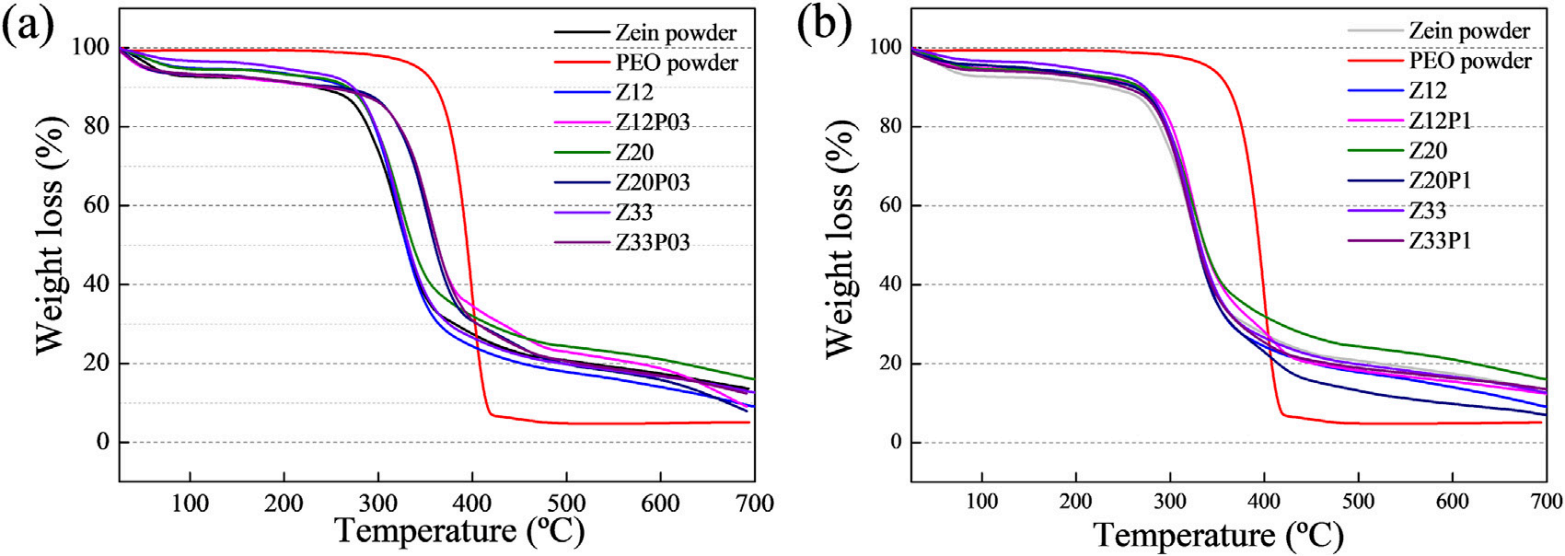
TG analysis of pure zein, pure PEO, and Z12, 20, and 33 with and without the addition of 0.3% PEO **(A)** and TG analysis of pure PEO, pure zein, and Z12, 20, and 33 with and without the addition of 1% of PEO **(B)**.

The thermogravimetric analysis (TGA) evaluated the thermal stability and the electrospun zein fibers with and without the PEO addition (Figure 7). All the samples show a similar profile and weight loss in three stages. The first is attributed to the loss of water molecules (up to 100°C). The second part shows the beginning of thermal degradation, and the last part shows the combustion zone. The first region of the graph showed similar behavior in moisture absorption except for pure PEO, which has hydrophobic behavior in graphs 7A–B. The second region of graph 7A–B, which declares the beginning of thermal degradation, shows that for both pure zein and mixtures of zein and PEO, the degradation temperature is at 270°C, which follows what was found in the literature (Medeiros et al., 2021). However, all blends have better thermal stability in terms of their percentage of mass loss when compared to pure zein, demonstrating the effect of PEO on the zein matrix. In Figure 7A, this behavior is more evident in the proportion of 0.3% PEO in zein. In the samples containing zein 20%/PEO 0.3% and Zein 33%/PEO 0.3%, there is an increase in the degradation temperature from 270°C to 287°C. The high degradation temperatures are a relevant characteristic of these fibers as they, based on the TG analysis, will probably allow them to be cooked at high temperatures without compromising their integrity. The fiber demonstrates unparalleled durability compared to conventional meat, which tends to undergo significant changes at high temperatures (Wolk, 2017).

This approach to producing plant-based meat fibers using zein fibers obtained by electrospinning with the addition of PEO can provide an innovative solution to improve the texture and appearance of these products. The results show that the formulation with 33% zein and 1% PEO (Z33P1) was the closest to the desired characteristics to mimic the fibers in animal meat. This solution was then used to add pea protein (PP) to the electrospun fibers. The interest in this protein is based on its well-balanced amino acid profile, low allergenicity, high commercial availability (Lam et al., 2018), and lower cost when compared to zein. However, this globular protein, in either its native or denatured state, does not form fibers because its molecules cannot exhibit sufficient entanglements or interchain associations (López-Rubio and Lagaron, 2012). Nevertheless, adding PP to the Z33P1 mixture led to a very high viscosity, making the electrospinning unfeasible. Therefore, the Z20P1 mixtures were chosen to add PP to produce electrospun fibers.

**FIGURE 8:**
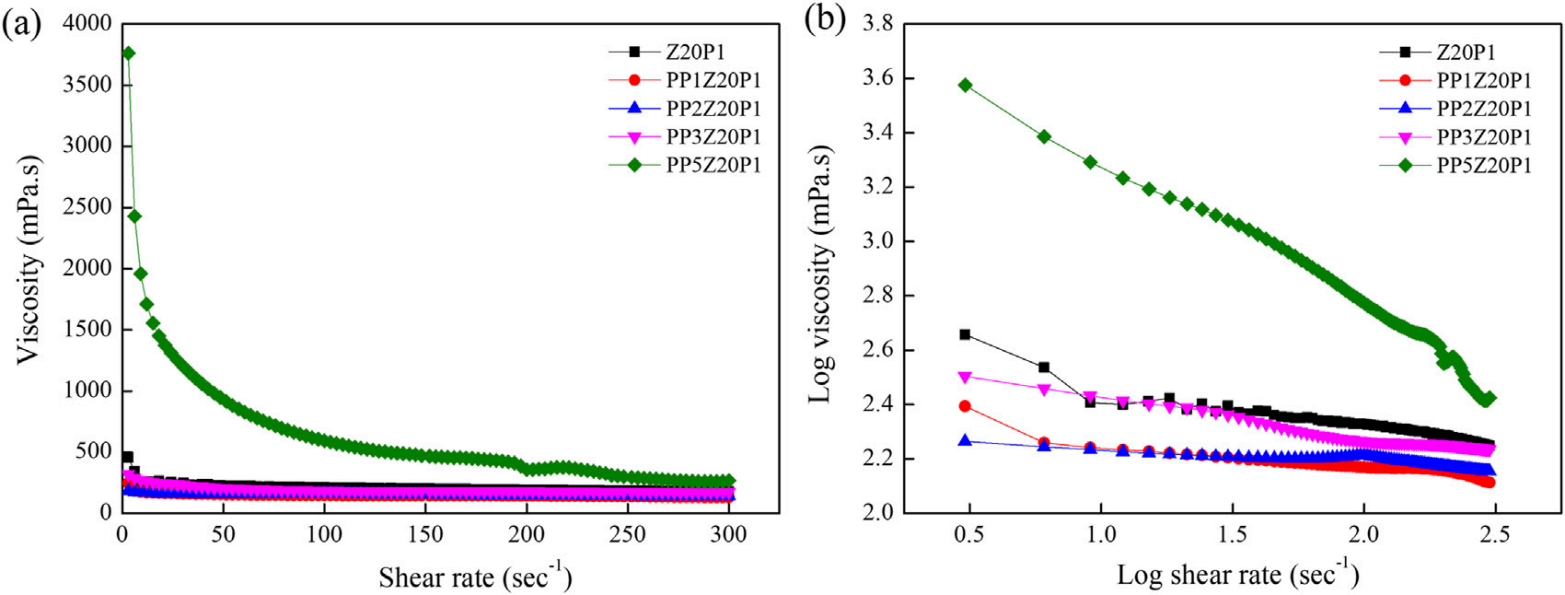
Viscosity as a function of shear rate **(A)** and log-log plot of the viscosity as a function of shear rate **(B)** Z20P1, PP1Z20P1, PP2Z20P1, PP3Z20P1 and PP5Z20P1 polymer solutions.

Figure 8 shows the flow curves of polymer solutions containing pea protein, zein, and PEO, and the rheological parameters are summarized in Table 2. The viscosity of Z20P1 and zein/PEO solutions with adding 1, 2, or 3 (%) of PP are similar, as shown in Figure 8, confirmed by the Tukey test presented in Table 2. However, increasing the PP concentration to 5% (w/v) increases the viscosity of the solution by approximately 3.8 times compared to the Z20P1 solution. The value of n is less than 1, which confirms that these samples exhibit shear-thinning behavior, as shown in Figure 8B. The Tukey test showed that the n values for the samples PP1% Zein 20% PEO1%, PP2% Zein 20% PEO1%, and PP3% Zein 20% PEO1% are similar. This result indicates that these samples present a nearly Newtonian-like flow behavior, while Zein 20% PEO1% and PP5% Zein 20% PEO1% present non-Newtonian behavior. The k value decreases when adding 1, 2, or 3% of PP. It increases with the addition of 5% of PP, indicating that the apparent viscosity decreases and increases again for a higher concentration of PP. The coefficient obtained (R^2^) varied from 96.96 to 99.99, which confirms that the Power law model used provided a good fit for the flow curves.

**TABLE 2:** Sample composition, sample designation, rheological parameters, mean of the results, the standard deviation, and the Tukey test for the rheological parameters for 80% ethanol aqueous solutions of different Pea protein/zein/PEO concentrations.

Sample composition (%)	Sample designation	k (Pa.s^n^)	n (−)	R^2^	Viscosity at 10 s^−1^ (mPa.s)	Fiber diameter (µm)
zein 20% PEO 1	Z20P1	0.5442^b^ ± 0.033	0.814^bc^ ± 0.003	99.93	286.9^b^ ± 23.7	8.34 ± 6.43
PP1%zein 20% PEO1	PP1Z20P1	0.218^b^ ± 0.016	0.893^a^ ± 0.010	99.89	158.0^b^ ± 1.41	3.98 ± 2.03
PP2%zein 20% PEO1	PP2Z20P1	0.317^b^ ± 0.005	0.884^a^ ± 0.001	99.99	275.0^b^ ± 5.53	7.29 ± 3.56
PP3%zein 20% PEO1	PP3Z20P1	0.287^b^ ± 0.011	0.940^a^ ± 0.013	99.90	318.0^b^ ± 61.2	7.39 ± 4.63
PP5%zein 20% PEO1	PP5Z20P1	1.816^a^ ± 0.804	0.658^b^ ± 0.128	96.96	1,135.0^a^ ± 152.23	7.29 ± 4.12

Equal letters indicate no significant difference between the means (Tukey test, p> 0.05) in the same column.

**FIGURE 9:**
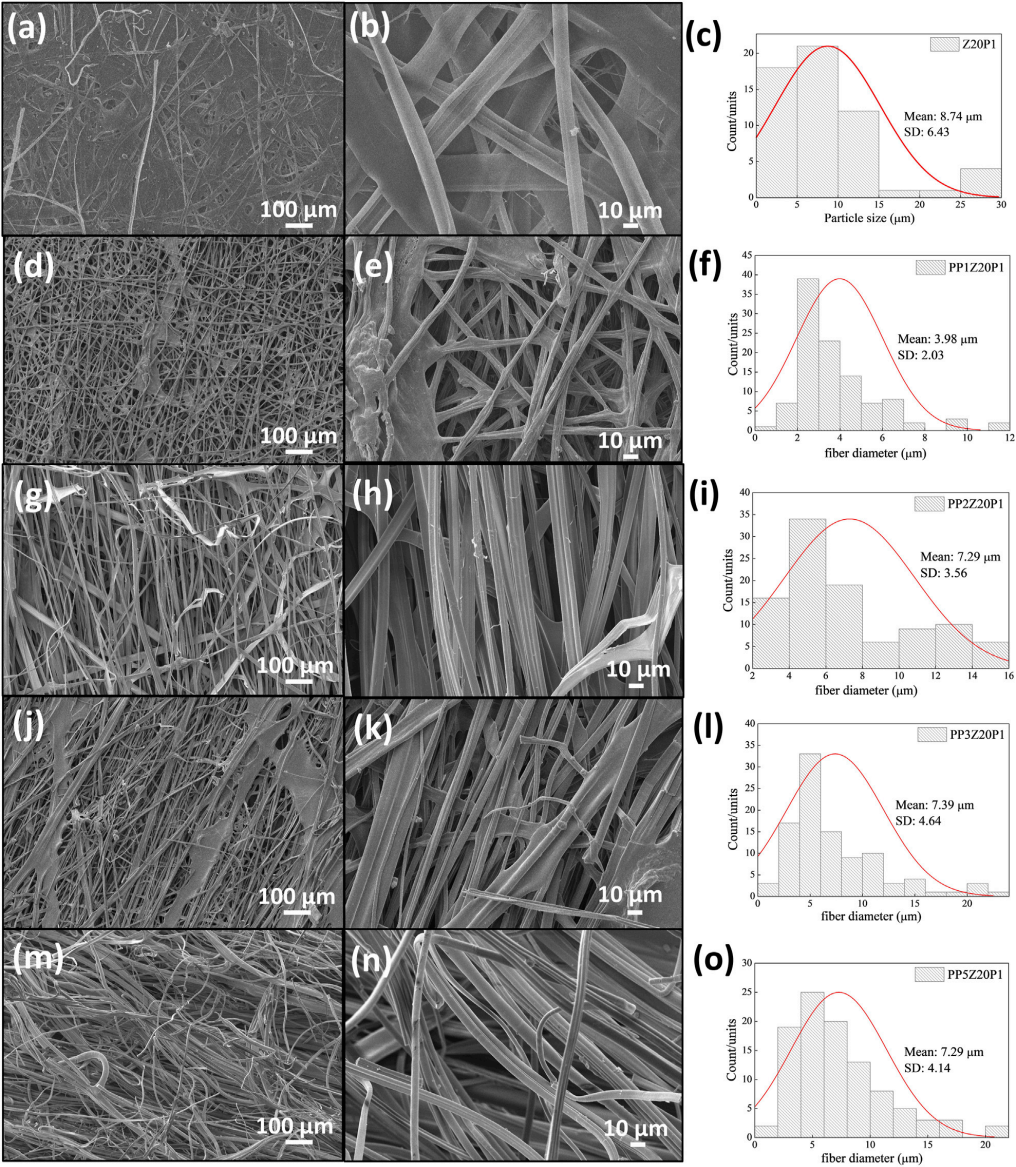
Z20P1 **(A, B)**, PP1Z20P1 **(D, E)**, PP2Z20P1 **(G, H)**, PP3Z20P1 **(J, K)** and PP5Z20P1 **(M, N)** and their fibers diameter **(C, F, I, L, O)**.

Figure 9 presents the SEM images of pea protein/zein/PEO fibers with different amounts of pea protein. The images showed that with the addition of 1% PP, the diameter of the fibers reduced by approximately 2.2 times when compared to the fiber without PP. However, with an increase in concentration above 1%, the diameter of the fibers remains almost unchanged (between 7.29 and 7.39 µm). One explanation for this behavior may be due to the increment of the solution viscosity since the fibers formed tend to be thicker and more irregular as the polymer molecules cannot stretch and align appropriately during electrospinning. As a result, the diameter of the fibers formed is practically unchanged, even with changes in the viscosity of the solution (Huang et al., 2003; Li et al., 2006).

**FIGURE 10:**
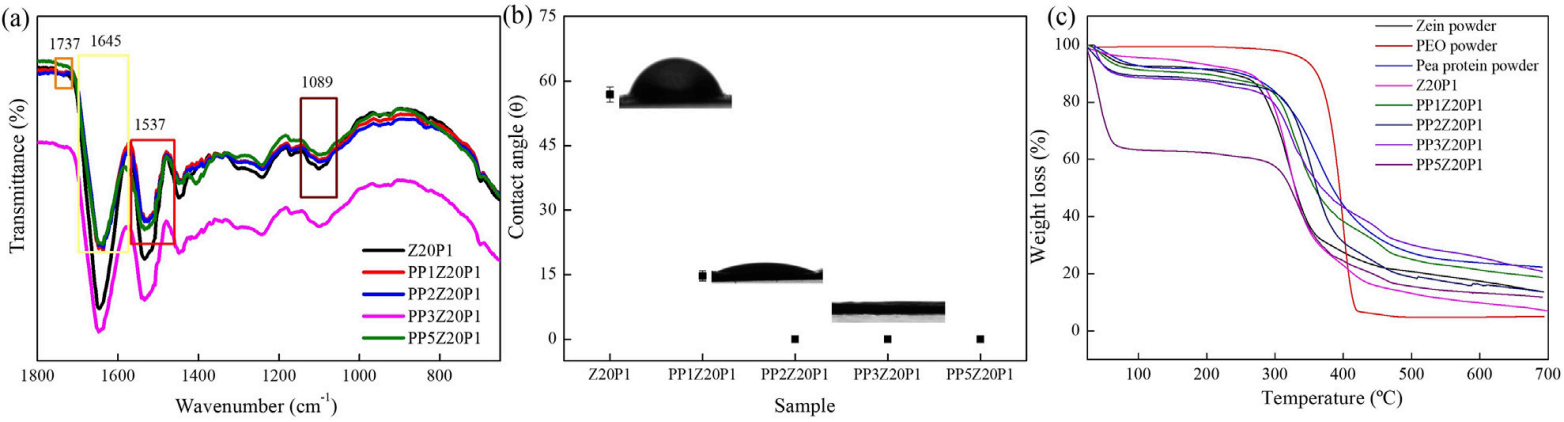
ATR-FTIR spectra **(A)**, water contact angle **(B)**, and TG curves **(C)** of Z20P1, PP1Z20P1, PP2Z20P1, PP3Z20P1, and PP5Z20P1 fibers.

Figure 10A shows the ATR-FTIR spectra of the electrospun fibers with different amounts of pea protein (PP) with zein/PEO (20:1). The band at 1737 cm^−1^, 1,645 cm^−1^, 1,537 cm^−1,^ and at 1,089 cm^−1^ refers to ester C = O stretching from triglycerides (Kaya-Celiker et al., 2015), amide I (Saxton and McDougal, 2021), amide II (Venyaminov and Kalnin, 1990), and carbohydrates (C-O stretch common to all polyhydroxy aldehydes and ketones) (Grube et al., 2002), respectively. All of them are the characteristic bands of pea protein and can be observed in all samples with this compound. They prove that PP was successfully incorporated into the zein/PEO fibers. However, as there is no formation of new absorption bands, we conclude that the interaction between PP/zein/PEO is only physical, i.e., no chemical reactions occur between them. The water contact angle, Figure 10B, indicates that PP addition makes the surface of the fibers more hydrophilic. With an increase in PP concentration above 1wt%, a contact angle equal to zero is observed. This behavior indicates that the surface of the fibers has complete or perfect wetting. A thermogravimetric analysis was conducted to evaluate the stability of zein fibers with the addition of pea protein (Figure 10C).

Two distinct phases of weight reduction were observed in all the recorded thermograms. The phase that starts around 30°C and ends at approximately 100°C was attributed to moisture loss. In the initial stage, about 8% of the mass of water was lost in the pea protein powder sample, indicating water evaporation. The second phase corresponds to the volatilization of protein fragments generated by decomposition reactions around 250°C (Ricci et al., 2018).

An increase in the PP amount in the PP/zein/PEO matrices increased the fibers’ hydrophilicity. Up to 100°C, the mass losses due to water absorption were 10%, 12%, 13%, and 35% for the samples (PP1Z20P1), (PP2Z20P1), (PP3Z20P1), and (PP5Z20P1), respectively. The contact angle results (Figure 10B) supported this significant increase.

The second phase of mass loss for all the PP fibers occurred between 285°C and 290°C. Compared to the zein/PEO fiber (Z20P1), which degrades at 270°C, the degradation temperature significantly increased when pea powder was added. This indicates that the addition of PP provides better stability to the fibers.

The results showed that combining pea protein with zein/PEO is a promising strategy to improve electrospun fibers’ formation capacity and quality. The combination of zein and pea protein can enhance the texture and palatability of the final product because of the greater hydrophilicity. With the hydrophilicity increased by adding PP, Figure 10B, the fiber’s water retention capacity increases, probably resulting in a juicier and softer final product when applied to processed food products. Moreover, the inclusion of pea protein enhances the thermal stability of the zein/PEO blend, indicating that this particular sample can endure high temperatures and, hypothetically, without adversely affecting its physical and sensory characteristics during cooking, Figure 10C.

The results showed that the exploration of incorporating pea protein into zein/PEO blends for electrospinning fibers presents a promising avenue for advancing plant-based meat alternatives. Future research can focus on optimizing the compatibility of other plant protein sources, such as soy and hemp, with biodegradable polymers to enhance the mechanical properties and texture of the resulting fibers. Additionally, incorporating natural additives like fibers or starches may improve the absorption of moisture and flavor, mimicking the sensory characteristics of animal-based products. However, several limitations must be addressed before widespread adoption can occur. These include challenges in achieving the desired fiber morphology and diameter for textural appropriateness, the variability in protein extraction processes that affect purity and functional performance, and potential issues with the scalability of electrospinning techniques in a commercial setting. Ultimately, overcoming these hurdles will require interdisciplinary collaboration, combining insights from materials science, food technology, and commercial production to fully realize the potential of electrospun plant protein fibers in the burgeoning market of plant-based meats.

## 4 Conclusion

The results showed the importance of using the zein protein and polyethylene oxide (PEO) to produce fibers for meat analogs by electrospinning method. The zein/PEO fibers at concentrations of 33% and 1%, respectively, demonstrated a significantly larger fiber diameter, which could contribute to a fiber more similar to that of traditional meat. Furthermore, this sample showed excellent hydrophilicity, which is essential to guarantee moisture retention and juiciness in vegetable protein-based products. Another aspect that can be highlighted is the good thermal stability of this combination. This means the Z33P1 sample can withstand high temperatures during processing and cooking without compromising its physical and sensory properties. By using zein as a source of vegetable protein, we guarantee a sustainable and healthy alternative compared to proteins of animal origin.

Furthermore, the combination of this zein/PEO blend with pea protein provides a solution to the reliability of pea protein. This combination, by having greater hydrophilicity, can improve the texture and palatability of the final product, creating an attractive fiber to be applied in plant-based meat analogs. Therefore, using pea protein with zein to produce electrospun vegetable meat brings significant advantages to health and the food industry, improving the quality of the final product, making it more attractive to consumers, and boosting the adoption of a more sustainable diet.

## Data Availability

The data that support the findings of this study are available from the corresponding author, ARCB, upon reasonable request.
